# Severe Fever with Thrombocytopenia Syndrome Acquired through Dog Bite, South Korea

**DOI:** 10.3201/eid3108.250090

**Published:** 2025-08

**Authors:** Uh Jin Kim, Hae Seong Jeong, Keon Kim, Ahrang Lee, Minji Kim, Sarah Kim, Sung Un Shin, Seung-Ji Kang, Sook In Jung, Hyungjun Kwak, Woong-bin Ro, Choon-Mee Kim, Dong-Min Kim, Kyung-Hwa Park

**Affiliations:** Chonnam National University Hospital, Gwangju, South Korea (U.J. Kim, H.S. Jeong, A. Lee, M. Kim, S. Kim, S.U. Shin, S.-J. Kang, S.I. Jung, K.-H. Park); Chonnam National University Medical School, Gwangju (U.J. Kim, S.-J. Kang, S.I. Jung, K.-H. Park); Chonnam National University College of Veterinary Medicine, Gwangju (K. Kim, W. Ro); Gwangsan-gu Public Health Center, Gwangju (H. Kwak); Chosun University College of Medicine, Gwangju (C.-M. Kim, D.-M. Kim); Chosun University Hospital, Gwangju (D.-M. Kim)

**Keywords:** severe fever with thrombocytopenia syndrome, viruses, Dabie bandavirus, zoonoses, transmission, dog, bite, South Korea

## Abstract

A veterinary technician in South Korea contracted severe fever with thrombocytopenia syndrome virus from a dog bite. Molecular evidence, including PCR sequencing, supports dog-to-human transmission. The case underscores the zoonotic risks posed by companion animals and highlights the importance of preventive measures.

Severe fever with thrombocytopenia syndrome (SFTS) is a zoonotic infectious disease caused by SFTS virus (*Dabie bandavirus*), primarily transmitted through tick bites (*1*). SFTS continues to spread across East Asia and poses a substantial public health threat; fatality rate in humans is ≈20% (*1*). Interspecies acquisition involving companion animals remains poorly understood; some reports suggest virus transmission from infected cats or dogs, but most lack definitive evidence such as documented bites (*2*–*4*). In South Korea, several canine SFTS cases have been reported (*5*). We describe a case of probable dog-to-human transmission of SFTSV through a bite, supported by molecular evidence.

A 23-year-old veterinary technician was transferred to Chonnam National University Hospital, a tertiary hospital in Gwangju, South Korea, after 6 days of fever. Laboratory findings showed leukopenia, thrombocytopenia, low C-reactive protein, and elevated liver enzymes and ferritin. The patient disclosed that a sick dog had bitten her right thumb 10 days before hospital admission; she had fed the dog wearing a mask but not gloves. The 7-mm wound had begun to heal after initial bleeding. The wound was rinsed under running water for <5 minutes before she arrived; hospital staff later applied antiseptic and administered a tetanus vaccine. Although the patient had no history of outdoor activity or known tick exposure, SFTS was suspected and subsequently confirmed by blood PCR. Her condition worsened on hospital day 2; we performed plasmapheresis on days 3 and 6 in the intensive care unit, leading to improvement.

The suspected source was a 4-year-old neutered male Pomeranian experiencing high fever, leukopenia, and thrombocytopenia, admitted to an animal hospital 10 days before the patient’s hospital admission. According to its owner, the dog had experienced 4 days of fever and anorexia; lethargy was first noted ≈22 days before hospital admission. After 2 weeks of supportive care, the dog fully recovered.

To investigate potential dog-to-human transmission of SFTSV, we tested samples from the patient and the dog by reverse transcription PCR and immunofluorescent assay as previously described (*5*,*6*). To confirm the quantitative PCR results targeting the small segment by using the careGENE SFTS Virus RT-PCR kit (Wells Bio, https://www.wellsbio.net), we performed nested PCR. The dog’s saliva (collected on patient’s hospital day 4) showed a low level of SFTSV RNA (cycle threshold value 36.44), although no band was visible on nested PCR (Table). Sequencing of the nested PCR amplicon from blood samples revealed 99.6% identity in the medium segment and 100% in the large segment (Figure). Virus culture and sequencing of the small segment were unsuccessful.

**Table:** Characteristics of samples infected with severe fever with thrombocytopenia syndrome virus, South Korea*

Source	Day	Specimen	Nested PCR				qPCR		IFA†	
			S seg, 346 bp	M seg, 540 bp	L seg, 860 bp		S seg, 71 bp		IgG	IgM
Human	HD 2	Blood	Positive	Positive	Positive		26.76		<1:32	<1:32
Dog	HD 0	Blood	Weak positive	Positive	Positive		32.99		ND	ND
	HD 2	Blood	Negative	Negative	Negative		38.32		>1:1,024	ND
	HD 4	Urine	Negative	Negative	Negative		32.18		1:32	ND
	HD 4	Saliva	Negative	Negative	Negative		36.44		ND	ND

*HD, hospital day. IFA, indirect immunofluorescence assay; L, large; M, medium; ND, not done; qPCR, quantitative PCR; S, small; seg, segment. †IFA was performed on serum samples for both the patient and the dog. For the dog, urine and saliva specimens collected on HD 4 (July 6) were also tested in an exploratory manner to assess potential antibody presence in nonserum body fluids.

**Figure:**
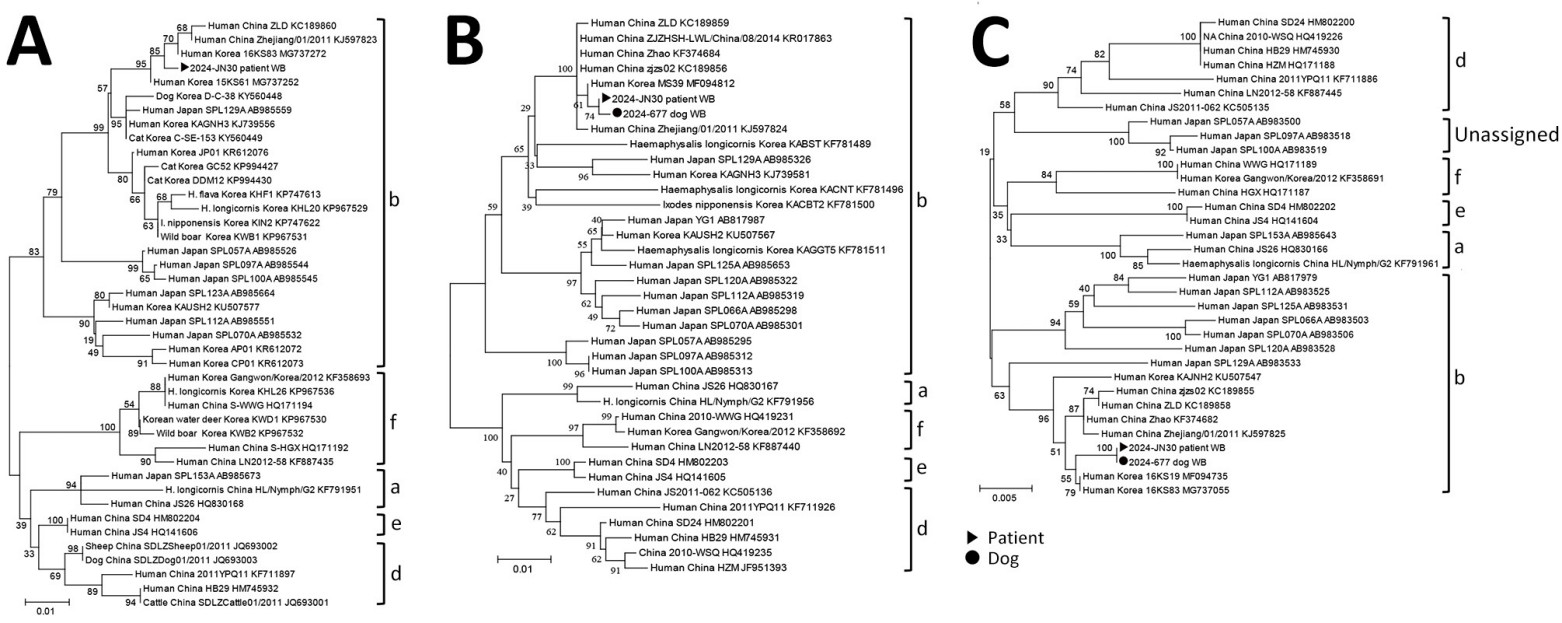
Phylogenetic analysis of SFTSV small (321 bp) (A), medium (477 bp) (B), and large (696 bp) (C) segments from human patient and dog, South Korea. Clustal X version 2.1 (http://www.clustal.org/clustal2) was used to construct the phylogenetic trees by using neighbor-joining with 1,000 bootstrap replicates. Genotypes of SFTSV are labeled (a, b, d, e, f). BLASTn (https://blast.ncbi.nlm.nih.gov) analysis revealed and nucleotide identity with reference SFTSV strain MF094812 of 99.44% (534/537) for the patient sample and 99.45% (541/544) for the dog sample; nucleotide identity with reference SFTSV strain MF094735 was 99.62% (785/788) for the patient sample and 99.63% (802/805) for the dog sample. The large segment nested PCR results showed 100% identity between the 2 samples. Scale bar indicates number of nucleotide substitutions per site. SFTSV, severe fever with thrombocytopenia syndrome virus.

Although the dog lived in an urban area, it was walked daily in nearby parks with dense vegetation. In the epidemiologic investigation, no ticks were found directly on the dog. We collected a total of 11 ticks from a suspected tick exposure site, a 94 m–high trail (34°58′15′′N, 127°33′51′′E) the dog and its owner frequently visited. The collected ticks included 6 adult female, 3 adult male, and 1 nymph *Haemaphysalis longicornis* ticks and 1 *Ixodes granulatus* nymph. All collected ticks tested negative for SFTSV.

A total of 43 persons, 3 household members with direct exposure to its saliva or bodily fluids and 40 veterinary staff, had contact with the dog. The Korea Disease Control and Prevention Agency recommended 2 weeks of symptom monitoring and PCR testing for those with direct contact. No PCR-positive or symptomatic cases were identified. None of the patient’s 31 hospital contacts experienced symptoms.

This case provides strong molecular evidence of dog-to-human SFTSV transmission from a bite. Unlike previous reports that relied solely on serologic findings (*2*,*7*), this case was supported by sequence identity and a documented bite. Although we could not isolate viable virus and the viral load in saliva was low, our findings suggest that canine saliva, particularly through dog bites, represents a potential transmission route, consistent with previous studies that detected SFTSV RNA in dog oral swab specimens (*8*) and isolated live virus at ≈10^6^ RNA copies/mL concentration from cat saliva (*9*). A limitation of this study is the inability to culture the virus, likely caused by delays in specimen collection. In addition, the absence of early saliva samples limits definitive confirmation of bite-mediated transmission, although the epidemiologic and molecular findings strongly support this route. Serologic testing was not performed for contacts, and asymptomatic cases may have gone undetected.

This case underscores the importance of personal protective equipment and infection control in both veterinary and human healthcare settings to prevent zoonotic transmission. Prompt wound care after animal bite or scratches, including washing with soap and running water for >20 minutes, can reduce the risk for infections such as B virus or rabies (*10*). Although the effectiveness of this approach against SFTS is unproven, the same principle may reduce the risk for other viral infections transmitted through animal bites or saliva exposure.

In conclusion, this case emphasizes the risk for SFTSV transmission not only via tick bites but also directly through bites from infected dogs. Enhanced awareness and preventive strategies in both veterinary and human healthcare settings are critical to mitigating the risks of SFTS.
